# Automated Breast Ultrasound (ABUS)-based radiomics nomogram: an individualized tool for predicting axillary lymph node tumor burden in patients with early breast cancer

**DOI:** 10.1186/s12885-023-10743-3

**Published:** 2023-04-13

**Authors:** Yu Chen, Yongwei Xie, Bo Li, Hua Shao, Ziyue Na, Qiucheng Wang, Hui Jing

**Affiliations:** Department of Ultrasound, Harbin Medical University Cancer Hospital, 150 Haping Road, Nangang District, Harbin, 150081 China

**Keywords:** Radiomics, Axillary lymph node (ALN), Automated Breast Ultrasound (ABUS), Breast cancer, Retraction phenomenon

## Abstract

**Objectives:**

Preoperative evaluation of axillary lymph node (ALN) status is an essential part of deciding the appropriate treatment. According to ACOSOG Z0011 trials, the new goal of the ALN status evaluation is tumor burden (low burden, < 3 positive ALNs; high burden, ≥ 3 positive ALNs), instead of metastasis or non-metastasis.

We aimed to develop a radiomics nomogram integrating clinicopathologic features, ABUS imaging features and radiomics features from ABUS for predicting ALN tumor burden in early breast cancer.

**Methods:**

A total of 310 patients with breast cancer were enrolled. Radiomics score was generated from the ABUS images. Multivariate logistic regression analysis was used to develop the predicting model, we incorporated the radiomics score, ABUS imaging features and clinicopathologic features, and this was presented with a radiomics nomogram. Besides, we separately constructed an ABUS model to analyze the performance of ABUS imaging features in predicting ALN tumor burden. The performance of the models was assessed through discrimination, calibration curve, and decision curve.

**Results:**

The radiomics score, which consisted of 13 selected features, showed moderate discriminative ability (AUC 0.794 and 0.789 in the training and test sets). The ABUS model, comprising diameter, hyperechoic halo, and retraction phenomenon, showed moderate predictive ability (AUC 0.772 and 0.736 in the training and test sets). The ABUS radiomics nomogram, integrating radiomics score with retraction phenomenon and US-reported ALN status, showed an accurate agreement between ALN tumor burden and pathological verification (AUC 0.876 and 0.851 in the training and test sets). The decision curves showed that ABUS radiomics nomogram was clinically useful and more excellent than US-reported ALN status by experienced radiologists.

**Conclusions:**

The ABUS radiomics nomogram, with non-invasive, individualized and precise assessment, may assist clinicians to determine the optimal treatment strategy and avoid overtreatment.

**Supplementary Information:**

The online version contains supplementary material available at 10.1186/s12885-023-10743-3.

## Background

Breast cancer is the most common malignant tumor and the main cause of cancer-related death among women [1]. The axillary lymph node (ALN) status determines the need for systemic therapy, the extent of surgery, reconstruction options, and the need for radiation therapy after mastectomy. Accurate assessment of ALN status plays an important role in breast cancer treatment and prognosis.

Based on preoperative assessment, patients without suspicious ALN metastasis by ultrasound (US) should have a sentinel lymph node biopsy (SLNB). Patients with positive ALN metastasis on US are candidates for ultrasound-guided fine needle aspiration (FNA) or core needle biopsy (CNB). For a negative FNA/CNB, SLNB is indicated. If FNA/CNB is positive, axillary lymph node dissection (ALND) is indicated unless neoadjuvant therapy is given. ALND is associated with several complications, including lymphedema (found in up to 25% of women after surgery), infection, shoulder motion restriction, and major vascular and nerve damage [2]. Besides, clinical practice has demonstrated that an important number of patients undergo a secondary ALND when SLNB displays major lymph node involvement [3]. Therefore, accurate preoperative noninvasive assessment of ALN status is essential.

According to the findings of the Z0011 trials, the American Society of Clinical Oncology updated clinical practice guidelines, ALND can be omitted in patients with breast cancer with 1–2 positive SLNs without a decrease in disease-free survival or overall survival [4, 5]. So, the new goal of the ALN status evaluation is tumor burden (low burden, < 3 positive ALNs; high burden, ≥ 3 positive ALNs), instead of metastasis or non-metastasis.

Ultrasound is a widely-used tool in assessing the ALN status preoperatively as it is non-invasive, radiation-free, real-time, rapid, and convenient. Previous studies have proved that axillary ultrasound (AUS) may provide valuable information relevant to ALN status in breast cancer [6]. However, AUS mainly obtains visual image information and focuses on the qualitative analysis of lesions, the diagnostic performances of axillary ultrasound to detect ALN involvement highly depends on the experience of radiologists [3]. Ahmed et al. [7] showed that 43.2% of patients with positive ALN metastasis on US had a low lymph node tumor burden (ALN metastasis < 3). It means that almost half of patients with ALN metastasis positive US assessments are exempt from ALND, which some researchers believe may constitute overtreatment for these patients [8]. AUS may not be a reliable predictor for nodal metastasis [6, 9]. In the age of precision medicine, a more effective and individualized method is urgent to resolve this problem.

As an emerging three-dimensional imaging technique, Automated Breast Ultrasound (ABUS), addresses the limitations of conventional handheld ultrasound (HHUS) and automatically scans the breast based on special high-frequency broadband sensors [10]. Several recent studies [11, 12] have shown that some unique features of ABUS, although they are also visual assessment and qualitative descriptions, may provide additional information for breast lesions. Specifically, retraction phenomenon appears as a satellite model around the lesion, with high sensitivity (80%–89%) and specificity (96%–100%) for breast cancer [13–15]. Radiomics extracts high-throughput quantitative features that may not be directly observable with the naked eye from single or multiple medical images. Radiomics has been more recently applied to distinguish benign malignant breast lesions [16], predict lymph node status [17, 18], and even evaluate treatment response [19]. According to the radiomics quality score proposed by Lambin et al. [20], ABUS images with standardized, repeatable, and high-resolution characteristics would be fit for radiomics analysis. However, to our knowledge, there is no ABUS-based radiomics study to differentiate ALN tumor burden. For patients with high tumor burden can assist in identifying what kind of initial axillary surgery can overlook the SLNB and undergo ALND specifically and assist in employing neoadjuvant chemotherapy or individualized adjuvant radiotherapy. For patients with low ALN tumor burden, unnecessary treatment, and potential complications due to surgery can be avoided.

The purpose of this study was to develop a radiomics nomogram integrating clinicopathologic features, ABUS imaging features and radiomics features from ABUS for predicting ALN tumor burden in early breast cancer**.**

## Methods

### Patient selection

Ethical approval for this retrospective study was obtained from our institutional review board, and informed consent was canceled. All the patients with breast cancer confirmed by pathology in our institution from November 2018 to January 2021 were selected. The inclusion criteria were as follows: (1) axillary US and ABUS examinations were performed before biopsy or resection; (2) the ALN status of the patients was clearly verified by pathology after SLNB/ALND; (3) breast lesion with a diameter less than 5 cm (stages T1 and T2).

The exclusion criteria were as follows: (1) no complete clinicopathological data or axillary US and ABUS images; (2) the patient had undergone anticancer therapy (radiotherapy or chemotherapy); (3) the ABUS image quality was poor with artifacts.

All patients included in this study were randomly divided into the training and test sets at a ratio of 7:3.

### Data acquisition

ABUS examination was performed by two well-trained technologists using the Invenia™ automatic breast ultrasound system with automatic 6 ~ 14 MHz linear broadband transformers (covering a volume of 15.4 × 17.0 × 5.0 cm) (Invenia™ ABUS, automatic breast ultrasound system, Ge Healthcare, Sunnyvale, the United States). The converter could be automatically moved in the scanning box. The thickness of each frame was 0.5 mm, and 330 images were collected axial. Patients were supine position with their arms fully raised to expose the breast. Meanwhile, a wedge-shaped cushion was placed under one side of the body that helps keep the breast stable with the nipple pointing towards the ceiling. A hypoallergenic lotion was distributed evenly over the breast with an additional amount over the area of the nipple. A disposable membrane was used to aid coupling and to uniformly compress the entire breast, enabling greater penetration, improving detail resolution at depth, and eliminating the creation of artifacts at the periphery. After the image collection was completed, all images were sent to the workstation for 3D reconstruction to obtain crown, horizontal and sagittal surface images. Radiologists can read all the image information in the workstation at any time for diagnosis. For the target tumor, diameter was measured as the largest diameter found on the axial plane of ABUS. ABUS imaging features were synthetically analyzed in three planes (axial, coronal and sagittal) using the Breast Imaging-Reporting and Data System (BI-RADS) lexicon, including margin (smooth, spiculated, angular or circumscribed), shape (regular or irregular), echo pattern (hypoechoic or complex), posterior acoustic feature (no change, enhance or decrease), calcification (no, macro or micro), orientation (horizontal or vertical), hyperechoic halo, retraction phenomenon. The above assessment was done by two radiologists (radiologist 1 and radiologist 2, with 9 and 15 years of experience in breast US) who were blinded the clinical and pathological information, and any differences were resolved through consultation.

The US-reported ALN status was obtained from the US reports, and axillary images including important features of suspicious lymph nodes were documented into the Picture Archiving and Communication Systems (PACS). It was retrospectively reviewed and verified by two radiologists (radiologist 1 and radiologist 2, with 9 and 15 years of experience in breast US). Axillary US features of lymph nodes used to assess suspicion for malignancy were as follows: (1) cortical thickness of 3 mm or greater; (2) longest/shortest axis ratio < 2; (3) absence of fatty hilum [21].

### Tumor segmentation and radiomics feature extraction

The three-dimensional region of interest (3D-ROI) was manually drawn around the boundary of the mass on the axial ABUS images by a radiologist with 5 years of experience in breast US using the SEG3D2 software (https://www.sci.utah.edu/cibc-software/seg3d.html).

**Fig. 1:**
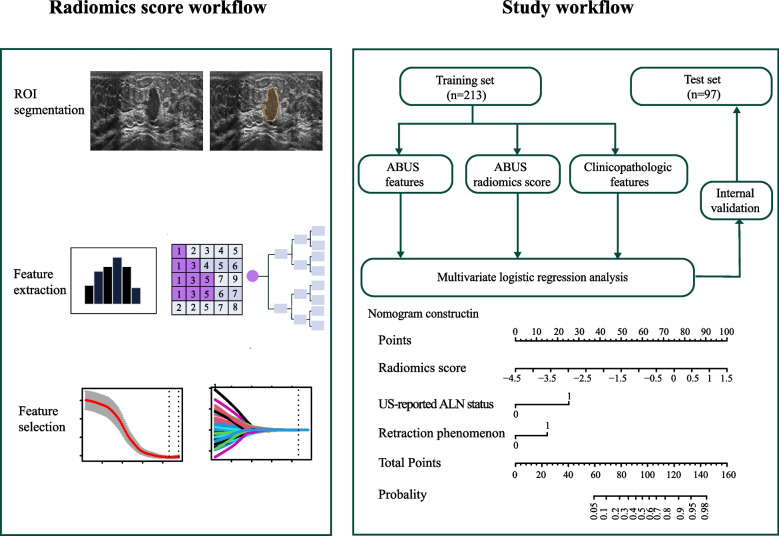
Radiomics score workflow and study flowchart. Tumor was manually drawn ROI from the axis ABUS images by using the SEG3D2 software. Next, 837 features, including first-order statistics, textural and wavelet features, were extracted by pyradiomics. ICC > 0.75, correlation coefficient ≥ 0.9, tenfold cross-test, and the LASSO regression were applied to data dimension reduction and select the most significant ALN tumor burden related radiomics features. Univariable and multivariate logistic regression analysis was used to develop the predicting model. We incorporated all independent predictors, and this was presented as ABUS radiomics nomogram

The first-order statistics, textural and wavelet features were extracted automatically from each ABUS image by pyradiomics (https://pyradiomics.readthedocs.io/en/latest/index.html). All the extracted features were in concordance with the standard set by the Imaging Biomarker Standardization Initiative (IBSI) [22]. Next, all radiomic features were rescheduled using Z-score normalization to facilitate subsequent statistical analysis. Figure 1 shows the flowchart of the radiomics score workflow and study flowchart.

To assess the inter-observer reproducibility, another radiologist (with 3 years of experience in breast US) drew 3D-ROI from 60 randomly chosen images. Intraclass correlation coefficient (ICC) was used to assess the inter-observer agreement, which was graded as excellent (0.90 to 1.00), good (0.75 to 0.90), moderate (0.50 to 0.75),or poor (< 0.50) [23]. The stable features with ICC > 0.75 were selected to adopt different segmentations.

### Radiomics score

We used Pearson correlation coefficient (normally distributed data) or Spearman’s rank correlation coefficient (nonnormal or rank data) to evaluate the redundancy of the features and eliminated redundant features with correlation coefficient ≥ 0.9, with only the one left for next analysis. Then, the least absolute shrinkage and selection operator (LASSO) regression using tenfold cross-test was applied to select the most significant ALN tumor burden related radiomics features.

### Model construction

Univariate and multivariate logistic regression analyses were performed to select the significant factors for ALN tumor burden. In univariate analysis, factors having *P* values < 0.10 were included in the multivariate analysis. Then, factors having *P* values < 0.05 were considered independent predictors after the multivariate analysis. Finally, ABUS model and radiomics nomogram was developed by incorporating these independent predictors.

### Model validation

In this study, the validity of the prediction model was assessed through Receiver operating characteristic (ROC) curve, calibration curve, and decision curve.

ROC curves were plotted to assess the performance of the prediction model for ALN tumor burden in the training and test sets. The relevant metrics, including areas under the curve (AUC), sensitivity, specificity, accuracy, positive predictive value (PPV), and negative predictive value (NPV), were also calculated.

Calibration curves were plotted to explore the predictive accuracy of the radiomics nomogram in the training and test sets. Besides, the goodness-of-fit was evaluated with the Hosmer–Lemeshow test.

To demonstrate the clinical value of the radiomics nomogram decision curves were drawn.

### Statistic analysis

Statistical analyses were performed using R software (version 4.1.2, https://www.r-project.org/). Categorical variables were compared using the chi-square test, and continuous variables were compared using the t-test or Man-Whitney U test to evaluate the consistency of the factors in the training and test sets. The reported statistical significance levels were all two-sided, and *p* values less than 0.05 were considered statistically significant. R software was used to construct and assess the radiomics score and the prediction model (details shown in Additional file 1).

## Results

### Basic information

A total of 310 patients were enrolled and randomly divided into a training set (*n* = 213) and a test set (*n* = 97) in this study. Table 1 showed the characteristics of breast cancer in training and test sets. There were 41(19.2%) and 17(17.5%) patients with ALN high tumor burden of early breast cancer in the training and test sets. There were no significant differences between the sets in the characteristics (*p* > 0.05).

**Table 1:** Characteristics of breast cancer in training and test sets

Characteristic	Training set(*n* = 213)	Test set(*n* = 97)	*P*-value
**Age, mean, years**	52.5 ± 10.0	55.0 ± 11.4	0.075^b^
**Diameter(cm)**	2.21 ± 0.84	2.22 ± 0.81	0.785 ^b^
**Histological type**			
Invasive ductal carcinoma	190(89.2)	83(85.6)	0.521^a^
Invasive lobular carcinoma	9(4.2)	4(4.1)	
Others	14(6.6)	10(10.3)	
**Estrogenic receptor (%)**			
Positive	170(79.8)	77(79.4)	0.930^a^
Negative	43(20.2)	20 (20.6)	
**Progesterone receptor (%)**			
Positive	143(67.1)	69(71.7)	0.483^a^
Negative	70(32.9)	28(28.3)	
**HER2 (%)**			
Positive	153(71.8)	65(67.0)	0.389^a^
Negative	60(28.2)	32(33.0)	
**Ki-67 status (%)**			
Positive (≥ 14%)	133 (62.4)	62(63.9)	0.803^a^
Negative (< 14%)	80(37.6)	35(36.1)	
**US-reported ALN status (%)**			
Positive	126(59.2)	50(51.5)	0.210^a^
Negative	87(40.8)	47(48.5)	
**Radiomics score**	-1.775 [-1.222, -2.168]	-1.831 [-1.142, -2.359]	0.766 ^b^
**ALN tumor burden**			
Low burden	172(80.8)	80(82.5)	0.718^a^
High burden	41(19.2)	17(17.5)	
**Margin**			
smooth	15(7.0)	8(8.2)	0.970^a^
spiculated	110(51.6)	48(49.5)	
angular	38(17.8)	17(17.5)	
indistinct	50(23.5)	24(24.7)	
**Shape**			0.165^a^
irregular	199(93.4)	95(97.9)	
regular	14(6.6)	2(2.1)	
**Echo pattern**			0.489^a^
hypoechoic	195 (91.5)	91(93.8)	
complex	18(8.5)	6(6.2)	
**Posterior acoustic features**			0.824^a^
no change	143(67.1)	62(63.9)	
enhance	34(16.0)	16(16.5)	
decrease	36(16.9)	19(19.6)	
**Calcification**			0.222^a^
no	103(48.4)	45(46.4)	
macro	10(4.7)	1(1.0)	
micro	100(46.9)	51(52.6)	
**Orientation**			0.443^a^
horizontal	182(85.4)	86(88.7)	
vertical	31(14.6)	11(11.3)	
**Hyperechoic halo**			0.754^a^
Yes	63(29.6)	27(27.8)	
No	150(70.4)	70(72.2)	
**Retraction phenomenon**			0.874^a^
Yes	88 (41.3)	41(42.3)	
No	125(58.7)	56(57.7)	

Low burden, < 3 positive ALNs; high burden, ≥ 3 positive ALNs

Data expressed as n (%), unless otherwise

Radiomics score was represented by median(interquartile)

^a^by the chi-square test, ^b^ by the Man-Whitney U test

*US* Ultrasound, *ALN* Axillary lymph node

### Radiomics score

**Fig. 2:**
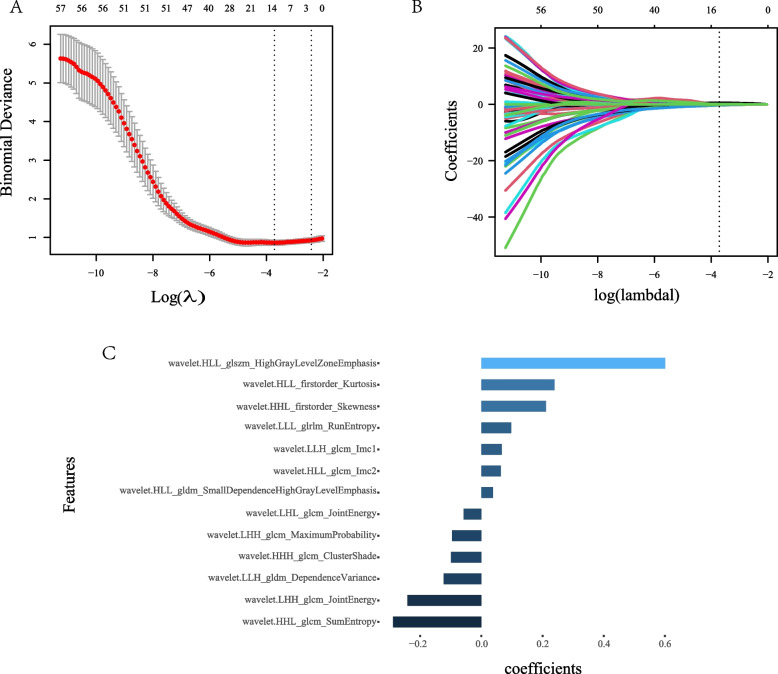
Radiomics features selection using tenfold cross-test and LASSO regression. **A **Tuning parameter(λ) selection in the LASSO regression used tenfold cross-test based on the minimum criterion. Dotted vertical lines indicated the optimal values using the minimum criteria and the1-SE criteria. A λ value of 0.024(log(λ) = -3.715) was chosen (minimum criterion) according to tenfold cross-test. **B** A coefficient profile plot was produced against the log(λ) sequence. Dotted vertical lines indicated the value obtained by the above tenfold cross-test, which resulted in 13 radiomics features with nonzero coefficients. **C** After tenfold cross-test and LASSO regression, the name and coefficient of selected radiomics features showed by a bar diagram

A total of 837 features (Additional file 2) were extracted from each patient’s axial ABUS images, including first-order statistics (*n* = 18), texture features (*n* = 75) and wavelet features (*n* = 738). First, in the evaluation of reproducibility, 508 showing ICC more than 0.75 were used for subsequent analysis. Second, we eliminated redundant features with correlation coefficient ≥ 0.9, and only one was chosen. Third, after the LASSO regression and tenfold cross-test, 13 radiomics features with nonzero coefficients were chosen (Fig. 2). Then, we got the final formula for radiomics score.

$$\text{radiomics score= -1.57486+wavelet.LLH\_glcm\_Imc1* 0.06655}{ } + \text{wavelet.LLH\_gldm\_DependenceVariance*-0.12317} \, \text{+wavelet.LHL\_glcm\_JointEnergy*-0.05780}$$

$$\text{+wavelet.LHH\_glcm\_JointEnergy*-0.24198}$$

$$\text{+wavelet.LHH\_glcm\_MaximumProbability *-0.09588}$$

$$\text{+wavelet.HLL\_firstorder\_Kurtosis *0.23917}$$

$$\text{+wavelet.HLL\_glcm\_Imc2*0.06358}$$

$$\text{+wavelet.HLL\_glszm\_HighGrayLevelZoneEmphasis*0.60089}$$

$$\text{+wavelet.HLL\_gldm\_SmallDependenceHighGrayLevelEmphasis*0.03775}$$

$$\text{+wavelet.HHL\_firstorder\_Skewness *0.21118}$$

$$\text{+wavelet.HHL\_glcm\_SumEntropy*-0.28907}$$

$$\text{+wavelet.HHH\_glcm\_ClusterShade*-0.09944}$$

$$\text{+wavelet.LLL\_glrlm\_RunEntropy *0.09735}$$

**Fig. 3:**
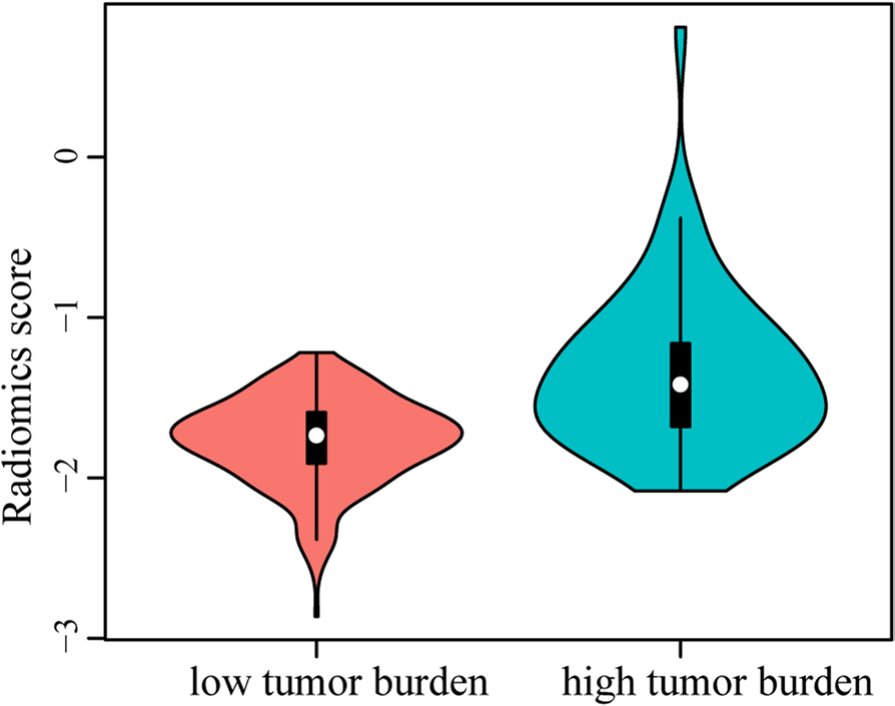
Distribution of radiomics score in high and low tumor burden patients. The patients with high tumor burden had significantly higher score than those with low tumor burden (*P* < 0.001)

The radiomics score of the high and low tumor burden was calculated using 13 radiomic features, respectively. The violin plot (Fig. 3) showed that radiomics score in the high tumor burden was significantly higher than low tumor burden (*P* < 0.001).

### Model construction

Univariable logistic regression analysis results showed in Table 2. On the multivariable analysis (Table 3) that included ABUS imaging features (the ABUS model), diameter (odds ratio [OR], 2.03, 95% CI: [1.292,3.256], *P* = 0.002), hyperechoic halo (OR, 2.48,95% CI: [1.149,5.388], *P* = 0.020), and retraction phenomenon (OR, 6.53, 95% CI: [2.972,15.542], *P* < 0.001) were independently associated with ALN tumor burden. On the multivariable analysis (Table 3) that included both clinicopathologic features, ABUS imaging features and radiomics score (the radiomics model), retraction phenomenon (OR, 3.34, 95% CI: [1.413,8.229], *P* = 0.006), US-reported ALN status (OR, 7.62, 95% CI: [2.429,33.904], *P* = 0.002), and radiomics score (OR, 3.82, 95% CI: [2.175.7.400], *P* < 0.001) were independently associated with ALN tumor burden.

**Table 2:** Results of univariable analysis in the training set

Characteristic	Odds Ratio	95% CI	*P*-value
**Age**	1.02	[0.986,1.055]	0.260
**Diameter**	1.71	[ 1.149.2.546]	0.008
**Histological type**			
Invasive ductal carcinoma	Reference		
Invasive lobular carcinoma	1.22	[0.177,5.312]	0.807
Others	1.16	[0.254,3.968]	0.820
**Estrogenic receptor**	1.05	[0.447,2.483]	0.905
**Progesterone receptor**	0.81	[0.400,1.660]	0.573
**HER2**	0.94	[0.442,1.982]	0.862
**Ki-67 status**	1.83	[0.859,3.888]	0.118
**Margin**			
smooth	Reference		
spiculated	4.12	[0.516,32.868]	0.182
angular	2.62	[0.288,23.886]	0.392
indistinct	3.07	[0.357,26.469]	0.307
**shape**	1.15	[0.581,2.289]	0.683
**Posterior acoustic features**			
no	Reference		
enhance	2.42	[1.013,5.784]	0.047
decrease	2.23	[0.942,5.301]	0.068
**Calcification**			
no	Reference		
macro	1.15	[0.583,2.278]	0.683
micro	1.10	[0.554,2.166]	0.794
**Orientation**	1.27	[0.506,3.188]	0.611
**Posterior acoustic features**			
no	Reference		
enhance	2.42	[1.013,5.784]	0.047
decrease	2.23	[0.942,5.301]	0.068
**Hyperechoic halo**	2.51	[1.244,5.074]	0.010
**Retraction phenomenon**	5.36	[2.508,11.459]	< 0.001
**US-reported ALN status**	12.09	[3.595,40.663]	< 0.001
**Radiomics score**	5.01	[2.829,8.856]	< 0.001

**Table 3:** Comparison of the multivariable models for ALN tumor burden in the training set

Characteristic	Beta Coefficient	Odds Ratio	95% CI	*P*-value
**ABUS model**				
Diameter	0.706	2.03	[1.292,3.256]	0.002
Hyperechoic halo	0.909	2.48	[1.149,5.388]	0.020
Retraction phenomenon	1.878	6.53	[2.972,15.542]	< 0.001
**ABUS nomogram**				
Retraction phenomenon	1.207	3.34	[1.413,8.229]	0.006
US-reported ALN status	2.031	7.62	[2.429,33.904]	0.002
Radiomics score	1.338	3.82	[2.175.7.400]	< 0.001

**Fig. 4:**
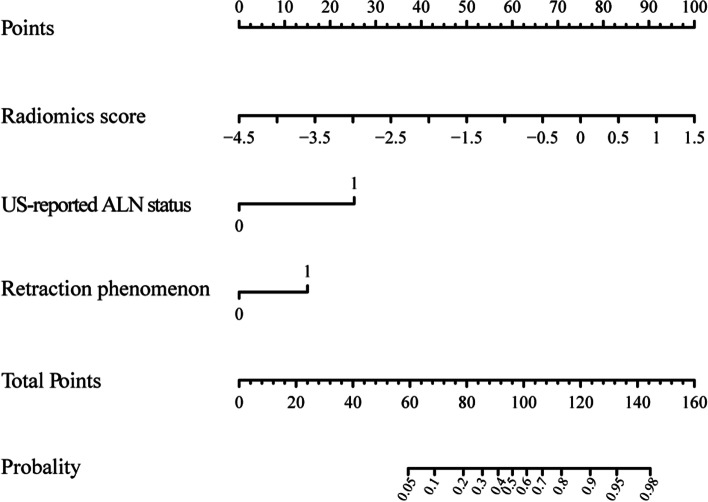
Developed ABUS radiomics nomogram. The ABUS radiomics nomogram was developed in the training set, incorporating the radiomics score, US-reported ALN status and retraction phenomenon. US, ultrasound; ALN, axillary lymph node

The radiomics nomogram that incorporated the above independent predictors was developed and presented as the nomogram (Fig. 4).

### Model validation

**Fig. 5:**
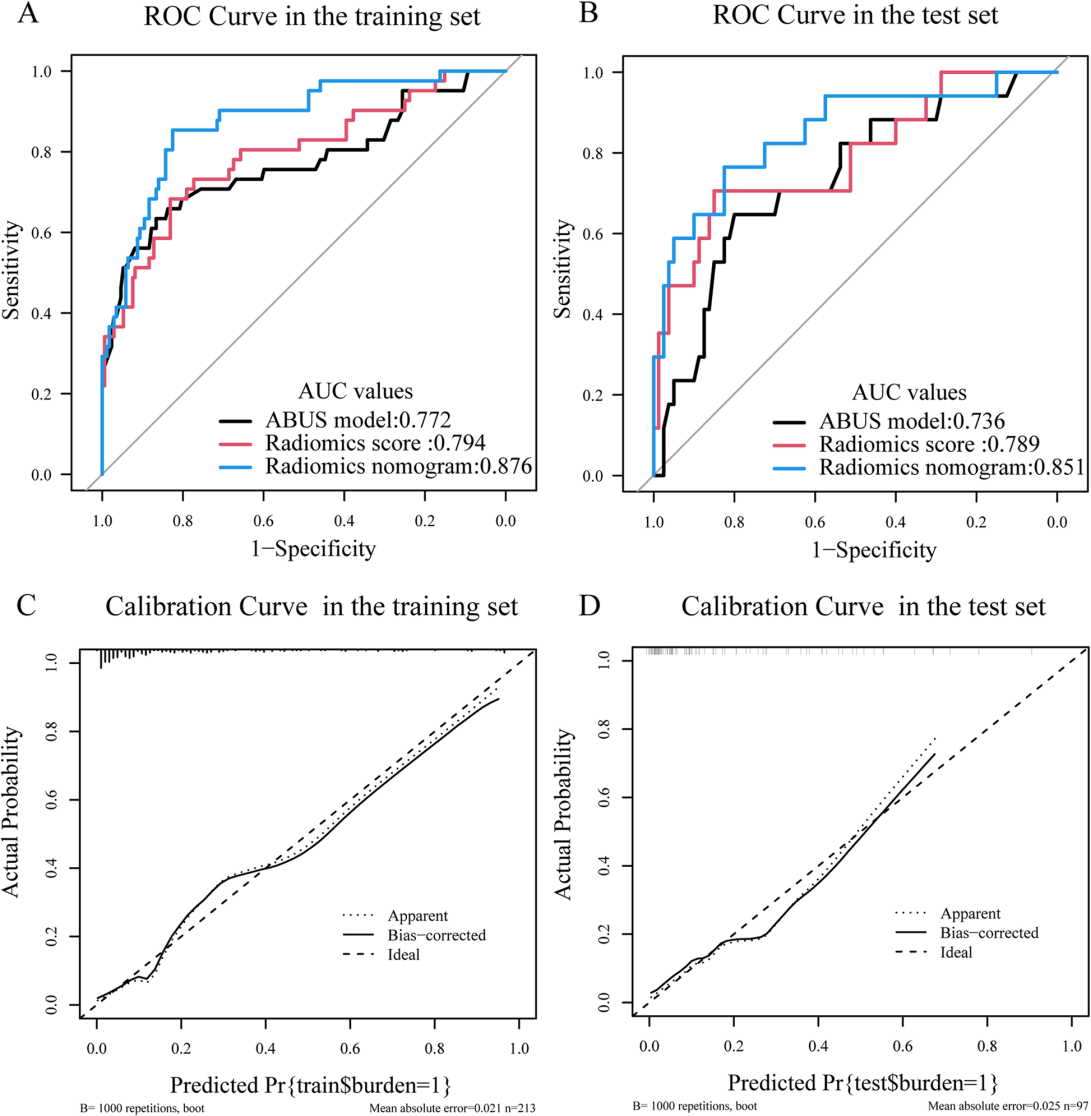
ROC Curves and Calibration curves of the model in the training and test sets. **A** ROC Curves of radiomics nomogram (blue, AUC:0.876), radiomics score (red, AUC:0.794), and ABUS model (black, AUC:0.772) in the training set. **B** ROC Curves of the radiomics nomogram (blue, AUC:0.851), radiomics score (red, AUC:0789), and ABUS model (black, AUC:0.736) in the test set. **C**, **D** Calibration curves of radiomics nomogram in the training (**C**) and test set (**D**). ROC, Receiver operating characteristic; US, ultrasound; ALN, axillary lymph node

Figure 5 A and B showed the performance of radiomics score, ABUS model, and radiomics nomogram for discriminating ALN status. The results of AUCs for radiomics score, ABUS model and radiomics nomogram were 0.794(95%CI:0.709,0.879), 0.772(95%CI:0.677,0.868),0.876(95%CI:0.815,0.937) in the training set and 0.789(95%CI:0.657,0.921),0.736(95%CI:0.602,0.870),0.851(95%CI:0.738,0.964) in the test set. Moreover, the performance of the radiomics nomogram was significantly more excellent than radiomics score and ABUS model both in the training and test sets (DeLong test *P* < 0.05). The diagnostic performance of radiomics score, ABUS model, and radiomics nomogram in the training and test sets was shown in Table 4. Furthermore, 200 times five-fold cross validation was performed to prove the robustness of radiomics nomogram in the training set, with a mean AUC of 0.863, a mean sensitivity of 0.861, a mean specificity of 0.831, and a mean accuracy of 0.839.

**Table 4:** The diagnostic performance of radiomics score, ABUS model, and radiomics nomogram in the training and test sets

Variables	Training set			Test set		
	**Radiomics score**	**ABUS model**	**Radiomics nomogram**	**Radiomics score**	**ABUS model**	**Radiomics nomogram**
AUC (95%CI)	0.794 (0.709,0.879)	0.772 (0.677,0.868)	0.876 (0.815,0.937)	0.789 (0.657,0.921)	0.736 (0.602,0.870)	0.851 (0.738,0.964)
Sensitivity	0.683	0.634	0.853	0.756	0.647	0.765
Specificity	0.831	0.866	0.826	0.850	0.800	0.825
Accuracy	0.803	0.822	0.831	0.825	0.773	0.814
PPV	0.491	0.531	0.538	0.500	0.407	0.481
NPV	0.917	0.909	0.959	0.931	0.914	0.943

*AUC* Area under the receiver operating curve

*95%CI* 95%confidence interval

*PPV* Positive predictive value

*NPV* Negative predictive value

The Calibration curves (Fig. 5 C and D) of the radiomics nomogram in the training and test sets showed an accurate agreement between the prediction of ALN tumor burden and pathological verification. The Hosmer–Lemeshow test showed X-squared = 5.926, *P* = 0.655(*P* > 0.05) in the training set and X-squared = 11.856, *P* = 0.158(*P* > 0.05) in the test set. It meant there is no significant difference between the predicted result and the actual outcome.

**Fig. 6:**
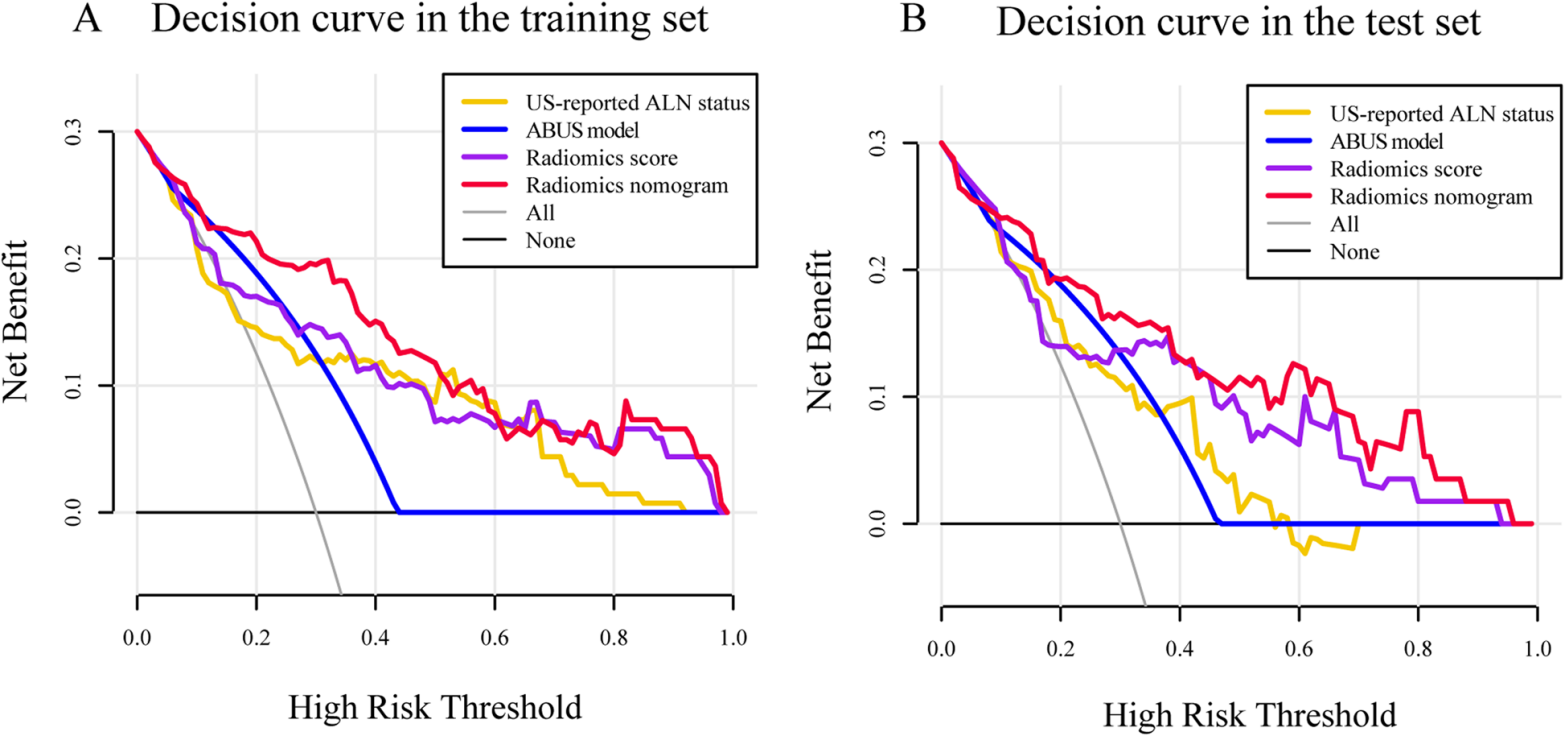
Decision curve of the radiomics nomogram (red line), radiomics score (purple line), ABUS model (blue line) and US-reported ALN status (yellow line) in the training set (**A**) and test (**B**) set. The vertical axis indicates the net benefit, the x-axis indicates the threshold probability. The black line indicates the presume that no patients showed ALN high burden, and the grey line indicates the presume that all patients showed ALN high burden

Decision curves (Fig. 6) were plotted to assess the clinical value of the different models in the training set and test sets. If the threshold probabilities are between 0.1 to 1, the radiomics nomogram will receive maximized net benefit.

To evaluate the value of the ABUS radiomics nomogram in making the optimal treatment strategies. We compared the performance of US-reported ALN status with radiomics nomogram in predicting ALN tumor burden (confusion matrix shown in Additional file 3). In our study, the false negative rates of radiologist and radiomics nomogram were 7.3% (3/41), 14.6% (6/41) in the training set, and 11.7% (2/17),23.5% (4/17) in the test set. The false positive rates of radiologist and radiomics nomogram were 51.1% (88/172),17.4% (30/172) in the training set, and 43.8% (35/80),17.5% (14/80) in the test set.

## Discussion

In the study, we constructed radiomics score based on ABUS image for predicting ALN tumor burden, which AUC values were 0.794 in the training set and 0.789 in the test set. The ABUS model, integrating diameter, hyperechoic halo, and retraction phenomenon, showed better performance, with AUC values of 0.772 in the training set and 0.736 in the test set. By multivariate logistic regression analysis, we developed a radiomics nomogram integrating the US-reported ALN status, retraction phenomenon and radiomics score, which showed best performance with AUC values 0.876 in the training set and 0.851 in the test set.

ABUS is a breakthrough in breast ultrasound that has been developed to meet the limitations of HHUS. ABUS provides three-dimensional imaging of the entire breast with multiplanar reconstructions, which has been demonstrated to improve diagnostic accuracy [24]. Several studies [11, 25–31] have focused on the ability of diagnosing breast lesions and assessment of response to neoadjuvant chemotherapy by ABUS. These studies indicated good diagnostic performance of ABUS. Recently, several studies [12, 32] showed some ABUS imaging features were significantly correlated with ALN status. In our study, ABUS model displayed adequate discriminative ability (AUC 0.772 in the training set and 0.736 in the test set). Three ABUS imaging features, diameter, hyperechoic halo, and retraction phenomenon, were obviously associated with ALN tumor burden (all *P* < 0.05), in this study. Some studies [33] reported that the size of primary breast cancer was significantly related to ALN status. Larger breast cancers have a wider range of glands invaded by cancer cells, and the probability of ALN metastasis through lymphatic drainage is also higher. In our study, retraction phenomenon was identified to be the strongest independent predictor with high diagnostic performance in differentiating high and low ALN tumor burden(*P* < 0.001). Desmoplastic reaction of breast malignancy, which can produce contraction of the surrounding tissues toward the mass and disrupt normal parallel tissue planes, might help explain the generation of retraction phenomenon [34]. The appearance of the retraction phenomenon indicates that breast cancer is highly aggressive and has poor response to treatment [35]. Jiang et al. [36] and Tang et al. [37]showed that the smaller and more superficial invasive carcinomas with lower histological grades tended to present with retraction phenomenon. A hyperechoic halo, also known as converging pattern, is caused by the compressed fibrous surrounding tissue or the infiltration between the tumor and the surrounding tissue [38, 39]. It reflects the degree of invasion of cancer cells and may be an important indicator of poor prognosis. Similarly, Tang et al. [37] reported that the malignant masses were associated with retraction phenomenon and discontinuous hyper-and hypoechoic rim (*p* < 0.001 for each).

However, visual assessment and qualitative descriptions for US features extremely rely on the personal clinical experience and subjective judgment of radiologists. Gillies et al. [40] have illustrated that tumor characteristics at the genetic and cellular levels can be captured from medical images by extracting and computing high-throughput features. Qiu et al. [41] extracted radiomics features from B-mode ultrasound and integrated the significant clinical characteristics of patients to construct radiomics model for predicting ALN metastasis. Therefore, this study comprised the radiomics signature and US-reported ALN status, with AUC values of 0.816 in the training cohort and 0.759 in the validation cohort. Similarly, Gao et al. [42] established a nomogram based on radiomics analysis of primary cancer B-mode ultrasound for predicting ALN tumor burden, with AUC values of 0.846 in the training cohort and 0.733 in the validation cohort. The results of above research were similar to our results. Jiang et al. [43] extracted radiomics features from shear-wave elastography and B-mode ultrasound and integrated Clinical characteristics of patients to construct radiomics model. The research showed in the performance of discriminating disease-free axillary (N0) and any axillary metastasis (N + (≥ 1)), it achieved a C-index of 0.845 for the training cohort and 0.817 for the validation cohort. The tool could also discriminate between low (N + (1–2)) and heavy metastatic ALN burden (N + (≥ 3)), with a C-index of 0.827 in the training cohort and 0.810 in the validation cohort.

ABUS provides standardized scanning protocols and uncouples detection from image acquisition, hence improving reproducibility, reducing operator dependency and radiologist workload [10]. To our knowledge, there is no published study that has showed whether the value of radiomics score from ABUS would benefit prediction of ALN tumor burden in early breast cancer. In our study, 13 radiomics features from axial images were screened out and used to established radiomics score. The ABUS radiomics score showed moderate discriminative ability (AUC 0.794 in the training set, 0.789 in the test set).

**Fig. 7:**
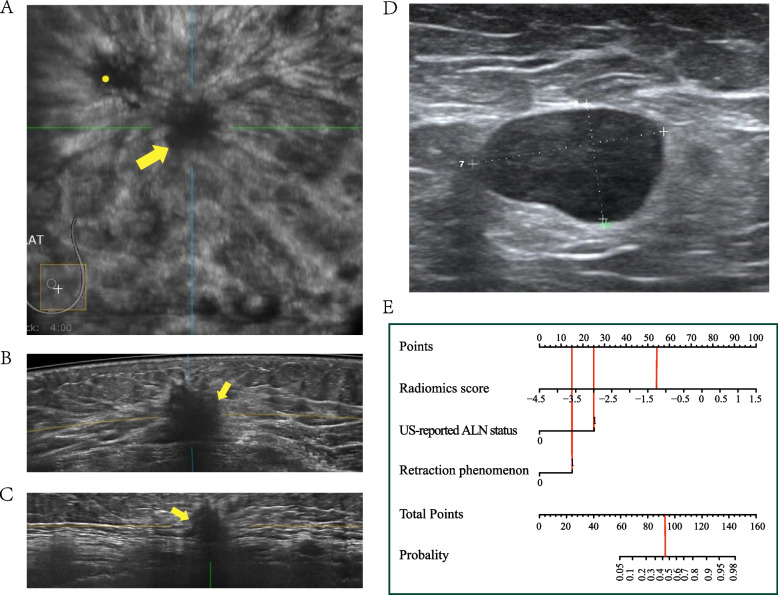
A case of radiomics nomogram. A 77-year-old woman who has a 3.1 cm diameter lesion, radiomics score = -1.301, retraction phenomenon positive and US-reported ALN positive, indicates a low burden, with a low probability of less than 45%. Pathology confirmed only one metastatic ALN in the patient. **A** coronal plane of ABUS examination. **B** axial plane of ABUS examination. **C** sagittal plane of ABUS examination. **D** Axillary US examination revealed suspiciously positive ALN (cortical thickening and lymphatic hilum disappeared). **E** The nomogram showed a low probability of high burden (< 45%), indicating ALN low tumor burden

We constructed a radiomics nomogram that integrated radiomics score with retraction phenomenon and US-reported ALN status to improve its predictive accuracy for ALN tumor burden. Based on the proposed risk classifier, the ABUS radiomics nomogram was able to classify patients into low- and high-tumor burden groups. According to ACOSOG Z0011 trials, we should pay more attention to the ALN tumor burden, instead of metastasis or non-metastasis. The radiomics nomogram resulted in 17.4% (30/172) and 17.5% (14/80) false positives in the training and test sets, which was obviously below the rates in US-reported ALN status (51.1% (88/172), 43.8% (35/80) in the training and test sets). The results showed that the radiomics nomogram could evidently reduce the false positive rate, compared with US-reported ALN status. Accurate forecasting of ALN high tumor burden can assist in identifying what kind of initial axillary surgery can overlook the SLNB and undergo ALND specifically and assist in employing neoadjuvant chemotherapy or individualized adjuvant radiotherapy [44]. Besides, for patients with low ALN tumor burden, unnecessary treatment, and potential complications due to surgery can be avoided. Figure 7 showed an example, the prediction of ALN tumor burden in patient.

Zhou et al. [45] firstly used deep learning methods in the evaluation of ALN status. Which included 974 primary tumor images of 756 patients with lymph node-negative early breast cancer, and validated the model in an independent validation set of 78 patients, with an AUC of 0.89. However, the logic behind the features and decisions based on deep learning is still a “black box”, which means the results based on deep learning are difficult to interpret [46]. On the contrary, in our study, “wavelet.LHH_glcm_JointEnergy”, “wavelet.HLL_firstorder_Kurtosis”, “wavelet.HHL_firstorder_Skewness”, and “wavelet.HHL_glcm_SumEntropy” were obviously associated with ALN tumor burden, indicating that these features strongly reflect ALN tumor burden. In our study, most of selected radiomics features are texture features after wavelet transform. Wavelet transform, which measures the resolution of image signals in different time, space, and frequency scale planes, is extremely useful for replaying even subtle but important texture information that is ignored by radiologists in low-contrast US images. Previous researches [19, 47] have demonstrated the texture features after wavelet transform are used to construct a prediction model.

There are several limitations to our study. First, the study was a retrospective study, inevitably there existed sample bias. Second, the sample originated from a single center and lacked external validation. Therefore, the number of positive patients was 58 (58/310,41/213 in the training set and 17/97 in the test set). The difference between the two sets may be caused by the small number of positive patients. In the future, more patients (particularly more positive patients), multi-center samples, and external validation are needed. Third, the three-dimensional ROI was manually delineated on the axis ABUS image by the radiologist. However, manually delineating the ROI was extremely time-consuming and inevitably involved inter-observer variability. In the future, we should research semi-automatic or automatic segmentation algorithms to overcome the above problems. Finally, we extracted radiomics features from intratumoral regions and failed to exploit peritumoral radiomics features in the study. The radiomics features of intratumoral and peritumoral regions should be integrated into further research.

## Conclusions

The ABUS radiomics nomogram showed favorable ability for predicting ALN tumor burden, which may provide additional benefits in treatment strategies for patients with early breast cancer, especially for patients with low tumor burden. With a more individualized and precise assessment for ALN tumor burden, radiomics nomogram will assist clinicians to make the optimal treatment strategy and avoid overtreatment.

## Supplementary Information

**Additional file 1: S1.** The details of R software used in this study.

**Additional file 2: Table S1.** The details of radiomic features.

**Additional file 3: Figure S1.** The confusion matrix of Radiomics nomogram and US-reported ALN status in the training and test sets.

## Data Availability

The datasets used and analysed during the current study available from the corresponding author on reasonable request.

## Abbreviations

ABUS: Automated Breast Ultrasound
ALN: Axillary lymph node
ALND: Axillary lymph node dissection
US: Ultrasound
SLN: Sentinel lymph node
SLNB: Sentinel lymph node biopsy
HHUS: Handheld ultrasound
3D-ROI: Three-dimensional region of interest
ICC: Intraclass correlation coefficient
BI-RADS: Breast Imaging-Reporting and Data System
LASSO: Least absolute shrinkage and selection operator
ROC: Receiver operating characteristic
AUC: Areas under the receiver operating characteristic curve
PPV: Positive predictive value
NPV: Negative predictive value
OR: Odds ratio
CI: Confidence interval
