# Supplementary material for: Automated Breast Ultrasound (ABUS)-based radiomics nomogram: an individualized tool for predicting axillary lymph node tumor burden in patients with early breast cancer
Source: BMC Cancer. 2023 Apr 13;23:340. doi: 10.1186/s12885-023-10743-3 (PMC10100322; doi:10.1186/s12885-023-10743-3)
Supplement: Supplementary file 1 — Additional file 1: S1. The details of R software used in this study. [file 12885_2023_10743_MOESM1_ESM.docx]

**S1** The details of R software used in this study.

The “irr” package was used for the inter-observer agreement. The “cor” function was used for the Pearson correlation coefficient and Spearman Rank Correlation Analysis and Pearson Correlation Analysis. The “glmnet” package was used for LASSO regression. The “glm” function was used for the univariate and multivariate logistic regression analyses. The “boxplot” function was used to plot the boxplot. The “pROC” package was used to plot the ROC curves and measure the AUCs, which were compared with DeLong’s test. The “vioplot” package was used to plot violin plot. The “calibrate” function of the “rms” package was used for the calibration curves. The “rmda” package was used to perform DCA.
