# Supplementary material for: Automated Breast Ultrasound (ABUS)-based radiomics nomogram: an individualized tool for predicting axillary lymph node tumor burden in patients with early breast cancer
Source: BMC Cancer. 2023 Apr 13;23:340. doi: 10.1186/s12885-023-10743-3 (PMC10100322; doi:10.1186/s12885-023-10743-3)
Supplement: Supplementary file 2 — Additional file 2: Table S1. The details of radiomic features. [file 12885_2023_10743_MOESM2_ESM.docx]

**Table S1** The details of radiomic features.

| **Radiomics feature** | Description |
| --- | --- |
| first-order statistics | distribution the distribution of voxel intensities within the ROI. |
| Gray Level Co-occurrence Matrix (GLCM) Features | distribution the second-order joint probability function of the ROI. |
| Gray Level Size Zone Matrix (GLSZM) Features | Quantification of gray level zones in the ROI (A gray level zone is defined as the number of connected voxels that share the same gray level intensity). |
| Gray Level Run Length Matrix (GLRLM) Features | Quantification of gray level runs in the ROI, which are defined as the length in the number of pixels, of consecutive pixels that have the same gray level value. |
| Neighboring Gray Tone Difference Matrix (NGTDM) Features | Quantification of the difference between a gray value and the average gray value of its neighbors within distance δ. |
| Gray Level Dependence Matrix (GLDM) Features | Quantification of gray level dependencies in an image. A gray level dependency is defined as the number of connected voxels within distance δ that are dependent on the center voxel. |
| Wavelet features | Wavelet filtering, yields 8 decompositions per level (all possible combinations of applying either a High or a Low pass filter in each of the three dimensions, including HHH, HHL, HLH, LHH, LLL, LLH, LHL, and HLL). |
