# Supplementary material for: Automated Breast Ultrasound (ABUS)-based radiomics nomogram: an individualized tool for predicting axillary lymph node tumor burden in patients with early breast cancer
Source: BMC Cancer. 2023 Apr 13;23:340. doi: 10.1186/s12885-023-10743-3 (PMC10100322; doi:10.1186/s12885-023-10743-3)
Supplement: Supplementary file 3 — Additional file 3: Figure S1. The confusion matrix of Radiomics nomogram and US-reported ALN status in the training and test sets. [file 12885_2023_10743_MOESM3_ESM.docx]

**Figure S1** the confusion matrix of Radiomics nomogram and US-reported ALN status in the training and test sets.

**
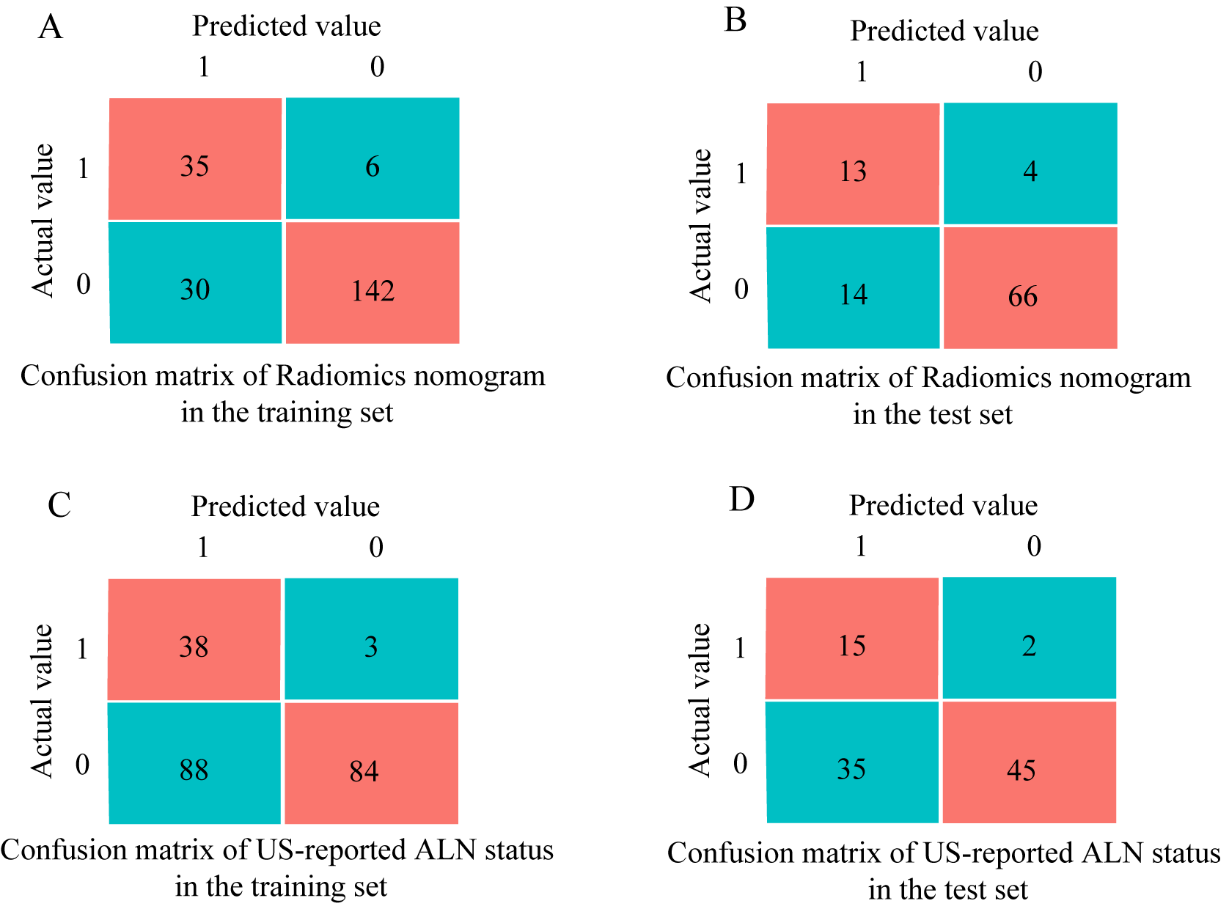
**
